# Evaluating amyloid-beta as a surrogate endpoint in trials of anti-amyloid-beta drugs in Alzheimer’s disease: a Bayesian meta-analysis

**DOI:** 10.57264/cer-2025-0095

**Published:** 2025-12-02

**Authors:** Sa Ren, Janharpreet Singh, Sandro Gsteiger, Christopher Cogley, Ben Reed, Keith R Abrams, Dalia Dawoud, Rhiannon K Owen, Paul Tappenden, Terrence J Quinn, Sylwia Bujkiewicz

**Affiliations:** 1 Sheffield Centre for Health & Related Research, School of Medicine & Population Health, University of Sheffield, Sheffield, UK; 2 Biostatistics Research Group, Division of Public Health & Epidemiology, School of Medicine, University of Leicester, Leicester, UK; 3 F. Hoffmann-La Roche Ltd, Basel, Switzerland; 4 Division of Public Health & Epidemiology, School of Medicine, University of Leicester, Leicester, UK; 5 Department of Statistics, University of Warwick, Coventry, UK; 6 National Institute for Health & Care Excellence, London, UK; 7 Faculty of Pharmacy, Cairo University, Cairo, Egypt; 8 Population Data Science, Swansea University Medical School, Swansea University, Swansea, UK; 9 School of Cardiovascular & Metabolic Health, University of Glasgow, Glasgow, UK

**Keywords:** Alzheimer's disease, amyloid-beta, clinical outcomes, meta-analysis, surrogate endpoint

## Abstract

**Aim:**

The use of amyloid-beta (Aβ) clearance to support regulatory approvals of drugs in Alzheimer’s disease (AD) remains controversial. We evaluate Aβ as a potential trial-level surrogate endpoint for clinical function in AD.

**Materials & methods:**

Data on the effectiveness of anti-Aβ monoclonal antibodies (MABs) on Aβ and multiple clinical outcomes were identified from randomized controlled trials through a literature review. A Bayesian bivariate meta-analysis was used to evaluate Aβ as a surrogate endpoint for clinical function across all MABs and for each individual anti-Aβ MAB. The analysis for individual therapies was conducted in subgroups of treatments and by applying Bayesian hierarchical models to borrow information across treatments.

**Results:**

We identified 23 randomized controlled trials with 39 treatment contrasts for seven MABs. The surrogate relationship between treatment effects on Aβ and Clinical Dementia Rating-Sum of Boxes (CDR-SOB) across all MABs was strong: with a meaningful slope of 1.41 (0.60, 2.21) and small variance of 0.02 (0.00, 0.05). For individual treatments, the surrogate relationships were suboptimal, displaying large uncertainty. Sharing information across treatments considerably reduced the uncertainty, resulting in moderate surrogate relationships for aducanumab and lecanemab. No meaningful association was detected for other clinical outcomes, including Mini Mental State Examination and Alzheimer’s Disease Assessment Scale-Cognitive Subscale.

**Conclusion:**

Although our results from the analysis of data across all MABs suggested that Aβ was a potential surrogate endpoint for CDR-SOB, individually the surrogacy patterns varied across treatments and showed no evidence of association. Bayesian information-sharing revealed moderate surrogate relationship only for aducanumab and lecanemab.

## Plain language summary

**What is this article about?:**

Some treatments for Alzheimer’s disease (AD) have been approved by regulators based on their ability to reduce levels of amyloid-beta (Aβ), a protein that builds up in the brains of people with AD. However, it remains unclear whether lowering Aβ actually leads to meaningful benefits for patients. In this study, we investigate whether changes in Aβ levels can reliably predict clinical benefit, using evidence from randomized controlled trials of anti-Aβ drugs.

**What were the results?:**

We found that changes in Aβ may be a good predictor of clinical benefits, measured by a test called Clinical Dementia Rating Scale-Sum of Boxes (CDR-SOB), which assesses thinking ability and daily functioning. This relationship was observed when looking at combined data from all anti-Aβ drugs. However, the association did not hold consistently for individual drugs. Using a statistical method that enables information sharing across drugs, we observed a moderate association between Aβ levels and clinical outcomes for the drugs aducanumab and lecanemab. Our findings also suggest that this relationship may vary between drugs and might not apply to new treatments.

**Why is this important?:**

The results from this study could provide valuable insight for decision-makers, such as NICE in England and Wales. The decision-makers often face limited evidence and have to rely on data from surrogate endpoints to make decisions whether a new treatment is likely to benefit patients and offer good value for money. This research may also contribute to a broader understanding of the surrogacy patterns in AD and inform future trials in this area.

Considerable research has focused on the development of monoclonal antibodies (MABs) aiming to inhibit the production of, or activate the clearance of, amyloid-beta (Aβ) in patients with Alzheimer’s disease (AD). This research has culminated in the completion of a number of randomized controlled trials (RCTs) assessing anti-Aβ drugs, including: aducanumab [1]; lecanemab [2]; donanemab [3] and gantenerumab [4], which vary in their mechanism of action. In particular, aducanumab and lecanemab select for soluble aggregated forms of Aβ, while other MABs target Aβ monomers (e.g., solanezumab), or do not discriminate between Aβ forms (e.g., bapineuzumab and crenezumab) [5,6].

The US FDA granted licensing approval for aducanumab, based on trial evidence demonstrating treatment efficacy in terms of reducing Aβ plaques in the brain, which was considered a surrogate endpoint for clinical benefit [7,8]. This generated controversy and criticism of FDA licensing approvals due to a lack of evidence that Aβ is an appropriate surrogate endpoint for clinical outcomes in AD [9–11]. Despite this controversy, another two FDA approvals were granted for lecanemab and donanemab [12]. More recently, EMA granted marketing authorization for lecanemab and donanemab, only after a re-examination process following earlier refusal due to limited effect on the cognitive function and concerns over side effects including amyloid-related imaging abnormalities (ARIA). Although a considerable reduction in Aβ has been observed for anti-Aβ MABs, there is lack of evidence of their long-term clinical benefit.

At the time of the FDA approval of aducanumab, only limited evidence existed about the association between Aβ and cognitive function, based on a relatively small cohort study [13]. However, no evidence was available about the association between the treatment effects on these two outcomes. Since then, several studies attempted to evaluate Aβ as a surrogate endpoint in AD [14–18]. Although these studies made valuable contribution to this research area, these evaluations were limited to either data from a single study [14] or meta-analyses of very few trials [15]. The wider meta-analysis [16–18] comprised of a mixture of drugs of different mechanisms of action utilizing an instrumental variable approach.

In this paper, we carry out a Bayesian meta-analysis of RCTs of anti-Aβ MABs to evaluate Aβ as a trial-level surrogate endpoint for clinical outcome. We evaluate surrogacy patterns between the treatment effects on the two outcomes across all MABs trials, and within subgroups of RCTs of individual MABs to acknowledge the potential impact of their different mechanisms of action on the surrogate relationships. We utilize a Bayesian framework to allow for borrowing of information on the surrogate relationship across treatments.

## Materials & methods

### Data sources, extraction & outcomes

We performed a literature search to identify recent systematic reviews of RCTs assessing the effectiveness of anti-Aβ MABs in patients with AD, followed by a search of the clinical trials database ClinicalTrials.gov. Our evidence base consisted of a comprehensive set of relevant RCTs, conducted in any phase, that reported treatment effects on both Aβ and clinical outcome, as included in the identified systematic reviews.

Data extraction from each trial was undertaken by two authors (SR and JS) and checked independently by two authors (CC and BR). Data were extracted from the corresponding trial publication, or from ClinicalTrials.gov when they were not reported in a trial publication. In cases where the treatment effect (and the associated standard error) was not reported numerically, these data were digitized from reported graphs using digitizer tools.

Data were extracted on the treatment effects on Aβ and clinical function. Data for the effect on Aβ were extracted on positron emission tomography (PET) standardized uptake value ratio (SUVR) as well as the Centiloid scale. Unreported data on one of the two scales for Aβ were imputed utilizing mapping equations identified from the literature [19–22], which are described in Appendix A. Treatment effects on clinical function were obtained for the following outcomes: Clinical Dementia Rating-Sum of Boxes (CDR-SOB), Mini Mental State Examination (MMSE), and Alzheimer’s Disease Assessment Scale-Cognitive Subscale (ADAS-Cog). The treatment effect on an outcome for each treatment contrast (MAB vs placebo) was defined as the difference in the change from baseline of the outcome measure between the active and control treatment arms. Data were extracted for the latest time point at which results were reported on both outcomes, and also at an earlier time point for Aβ and a later time point for clinical function if available.

### Statistical analyses

We adopted a Bayesian bivariate meta-analysis model (allowing for the incorporation of multi-arm trials) to perform trial-level evaluation of Aβ as a surrogate endpoint for clinical function, following the method of Daniels and Hughes [23]. The surrogate relationship between the treatment effects on Aβ level and on the clinical function was described in the form of a regression equation. The strength of the association was evaluated based on the criteria set out by Daniels and Hughes, who consider a perfect surrogate relationship when the intercept is zero, meaning that a null effect on the surrogate endpoint should imply a null effect on the final clinical outcome; slope is nonzero, which signified evidence of an association between effects on the surrogate endpoint and the final outcome, and the variance (of the treatment effect on the final outcome conditional on the effect on the surrogate endpoint) is zero, implying that the treatment effect on the final outcome could be predicted perfectly from the effect on the surrogate endpoint.

The Bayesian bivariate meta-analysis model developed by Daniels and Hughes has been specifically designed for trial-level surrogate evaluation [24]. This approach models the relationship between treatment effects on the surrogate endpoint and the final outcome, enabling prediction of the treatment effect on the final outcome from the effect on the surrogate endpoint while accounting for the uncertainty around the treatment effects on surrogate endpoints. It is, therefore, one of the methods that enables surrogate endpoint evaluation at the highest level of evidence, by assessing whether the technologies that improve the surrogate endpoint also improve the final outcome across many randomized controlled trials [25,26].

As a base case analysis, we assessed the surrogate relationship across all trials in the dataset, irrespective of treatment. Although the included treatments are all anti-Aβ MABs, their individual mechanisms of action differ. These drugs reduce Aβ accumulation through multiple pathways, including inhibition of plaque formation, inhibition of fibril extension, and facilitation of aggregate clearance, which may contribute to differences in their effectiveness [27]. The degree of impact of different mechanism of action on the effectiveness may vary across treatment and outcomes and therefore this may also impact the surrogate relationships. Such differences can influence how treatment effects on Aβ are translated into clinical benefits. Health technology assessment (HTA) agencies, such as NICE, often focus on a specific drug when evaluating the evidence base, including surrogate endpoints. As a result, understanding surrogate relationships for individual treatments is of particular interest to policy makers. In this paper, we first assessed the trial-level surrogate relationship for each treatment separately by carrying out subgroup analyses. Then we extended the analyses for individual treatments utilizing Bayesian hierarchical models [28]. The method allows for borrowing information about the surrogate relationships across treatments while retaining the ability to differentiate between the strong and weak surrogate association patterns for the individual treatments. Both full exchangeability and partial exchangeability hierarchical models have been adopted for different strength of borrowing [28]. We performed a range of sensitivity analyses by selecting different measures of treatment effect on clinical function and Aβ, including outcomes reported at different follow-up time, and considering the impact of the choice of prior distribution on the conditional variance. We performed leave-one-out cross validation to assess the predictive value of Aβ effect on the effect of clinical function [24].

Within-study correlation between Aβ and clinical function is needed for each study to populate the bivariate meta-analytic model; however, this was only reported for two RCTs [29] at the arm level. Obtaining within-study correlations is often challenging [30,31]. Daniels and Hughes recommended applying a common value when there is no available information for each study [23]. Where information was only available from a single trial, previous authors have imputed this value for the remaining studies [31–34]. Following this existing approach, we used correlations from the two trials with available data and applied these estimates across the remaining studies.

We implemented the models using the WinBUGS 1.4.3 software to estimate model parameters using Markov chain Monte Carlo (MCMC) simulation [35]. The WinBUGS code used in the analysis was adapted from the existing code published for the hierarchical model [28] by incorporating multi-arm studies. The results are presented as means with 95% credible intervals (CrIs). Additional analyses, including data management and graphics, were conducted using R software [36].

## Results

### Literature review

We identified 13 meta-analyses seeking to synthesize evidence from RCTs on the efficacy of anti-Aβ drugs for AD [37–49]. These meta-analyses focused on pooling effectiveness data on each outcome individually. Three of them synthesized the effects of anti-Aβ drugs on both PET Aβ and clinical outcome measures (ADAS-Cog, CDR-SOB and MMSE) [37–39]. Although the results of these meta-analyses showed an overall significant effect in reducing Aβ, the effect on the clinical outcome measures varied across the meta-analyses. There were six meta-analyses [40–45] synthesizing the treatment effects on clinical outcomes but not PET Aβ. One review reported treatment effects on PET Aβ but not on clinical outcomes [46]. Additionally, there were three systematic reviews comparing the efficacy of different MABs on various outcome measures using network meta-analysis [47–49]. Some further details pertaining to the conclusions of these meta-analyses can be found in Appendix B. Following detailed review of the identified meta-analyses, we found the systematic review by Jeremic *et al.* [45] most up to date and complete, as it comprised all relevant trials included in all the other systematic literature reviews. Thus, the trials identified by Jeremic *et al.* [45], formed the evidence base for our research. Our literature search also identified a review of ongoing phase II/III trials assessing the efficacy of anti-Aβ drugs in AD [50]. A review of these trials along with a search of ClinicalTrials.gov data base, which were conducted to ensure our evidence base was up to date, did not identify any additional completed trials.

#### Dataset

**Figure 1.**
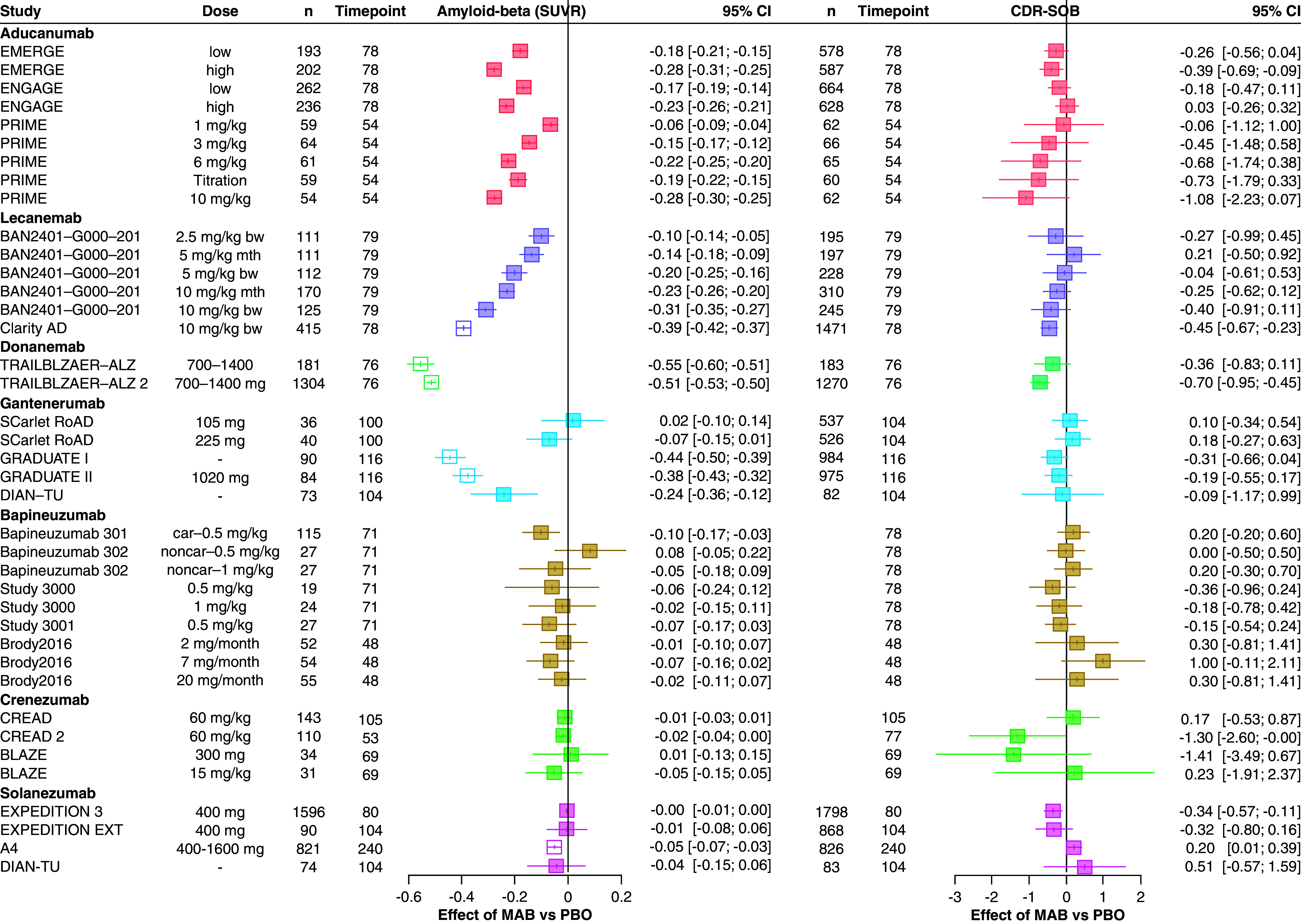
Forest plot illustrating the treatment effects of anti-amyloid-beta monoclonal antibodies on amyloid-beta levels (measured via PET) scan, on the standardized uptake value ratio scale and the Clinical Dementia Rating-Sum of Boxes outcome. The treatment effects represent the difference in change from baseline between MAB and PBO. Estimates shown as empty squares (no filled color) were imputed by applying a conversion formula based on the radioactive tracer used in the PET scan, for trials where the effect on amyloid-beta was reported on the Centiloid scale alone. CDR-SOB: Clinical Dementia Rating-Sum of Boxes; MAB: Monoclonal antibodies; PBO: Placebo; SUVR: Standardized uptake value ratio.

Figure 1 presents the dataset used to perform the meta-analysis evaluating the surrogate relationships. These data represent the observed effects on Aβ PET and CDR-SOB, comparing the active treatment arms with the placebo control arm in each trial. The effects are grouped by treatment. The data were obtained from 23 trials and 39 treatment contrasts reporting effects on both Aβ PET and CDR-SOB. Characteristics of included clinical trials can be found in Supplementary Table 1 of Appendix B.

For aducanumab, lecanemab and donanemab, trials demonstrated statistically significant effects on Aβ PET across different doses. There was evidence of a dose-response relationship, with a higher dose corresponding to a larger effect. However, for most of these treatments' doses there was no evidence of a statistically significant effect on CDR-SOB (exceptions were the high-dose arms in the EMERGE trial, Clarity AD trial and TRIALBLAZER-ALZ 2). Despite this, the point estimates indicate that a larger effect on Aβ PET is associated with a larger effect on CDR-SOB. There was larger evidential uncertainty for the other treatments (gantenerumab, bapineuzumab, crenezumab, solanezumab), where a statistically significant effect was apparent for five (out of 22) treatment contrasts on Aβ PET and for two (out of 22) treatment contrasts on CDR-SOB. Consequently, it is unclear whether there is an association between the effects on Aβ PET and CDR-SOB for these treatments.

### Surrogate endpoint evaluation

#### Surrogate relationships across all trials of MABs

Data from 23 identified studies with 39 treatment contrasts reporting the treatment effects on both Aβ and CDR-SOB were included. They comprised 14 two-arm studies, six three-arm studies, one four-arm study and two six-arm studies.

**Figure 2.**
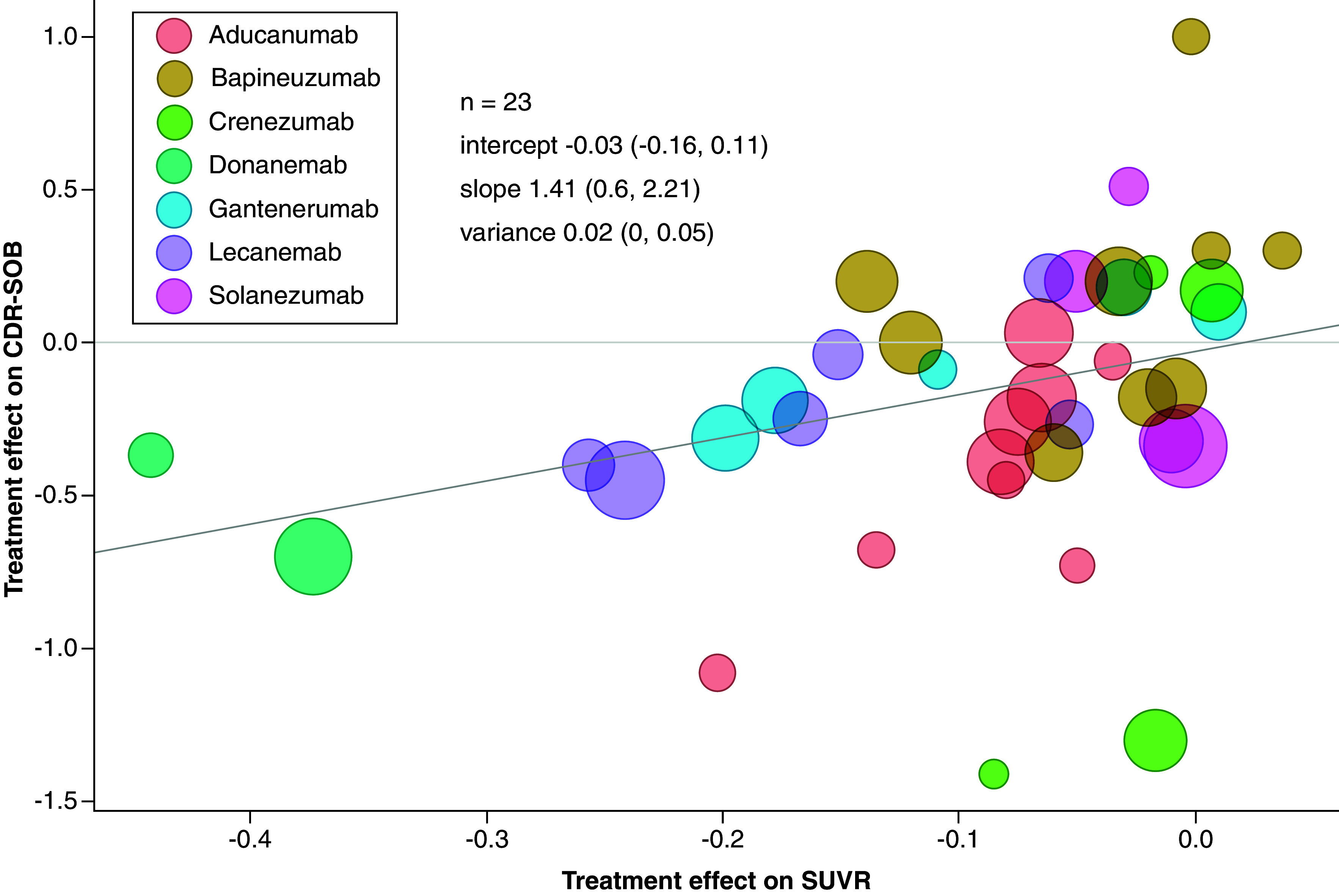
Bubble plot of the surrogate relationship between treatment effects on amyloid-beta measured on the standardized uptake value ratio scale and Clinical Dementia Rating-Sum of Boxes with treatment effects on amyloid-beta SUVR reported at earlier time points. The mean estimates (95% credible intervals) of intercept, slope and conditional variance were obtained from Daniels and Hughes model. The bubble size corresponds to the number of patients with CDR-SOB reported. CDR-SOB: Clinical Dementia Rating-Sum of Boxes; SUVR: Standardized uptake value ratio.

Figure 2 shows a bubble plot representing reported treatment effects on Aβ and CDR-SOB from all included studies with bubble size corresponding to the number of patients with reported CDR-SOB. The regression line represents the surrogate relationship between treatment effects on Aβ and CDR-SOB from the analysis of data across all MABs trials. The relationship across all MABs was strong, characterized by an intercept close to zero at -0.03 (95% CrI: -0.16, 0.11), a positive slope of 1.41 (95% CrI: 0.60, 2.21) and a small conditional variance of 0.02 (95% CrI: 0.00, 0.05).

Leave-one-out cross-validation was performed to evaluate the predictive value of Aβ as a surrogate endpoint for CDR-SOB. The cross-validation across all trials showed a good coverage rate with 95% of the predicted intervals including the observed estimates of the effects on CDR-SOB. A forest plot showing the observed effects and the predicted effects of CDR-SOB for each study can be found in Supplementary Figure 1 of Appendix C1.

Sensitivity analyses were conducted to further explore the surrogate relationships across all MABs, utilizing different measures of the treatment effect on Aβ and the clinical function. Two clinical outcomes were considered: ADAS-Cog and MMSE. When using ADAS-Cog as the measure of clinical function, data from 20 trials and 31 treatment contrasts were available. The surrogate relationship between treatment effects on Aβ SUVR and ADAS-Cog was estimated to be weak with much larger conditional variance, 0.06 (95% CrI: 0.00, 0.23), compared with the analysis using CDR-SOB as the final clinical outcome. The analysis of data on the clinical function measured by MMSE included 13 trials and 22 contrasts. We found lack of evidence of surrogate relationship between treatment effects on Aβ SUVR and MMSE, with the 95% CrI for the slope including both positive and negative values. Both sets of results are presented in Supplementary Figures 2 & 3 of Appendix C2. Our results suggest differences in surrogate relationships across clinical outcome measures, potentially due to variations in the measures themselves or in data consistency. We identified fewer studies reporting treatment effects on ADAS-Cog and MMSE than on CDR-SOB, and this limited evidence may have contributed to weaker or more uncertain surrogate relationships. These findings are further explored in the discussion section.

Another set of analyses considered outcomes reported at different follow-up times. The main analysis, reported above, included data with the follow-up at the earliest reported treatment effect on Aβ. When using the treatment effects on Aβ reported at the same time points as the clinical outcome, the surrogate relationship between treatment effects on Aβ SUVR and CDR-SOB was also strong, as presented in Supplementary Figure 4 of the Appendix C2. The surrogate relationship between treatment effects on Aβ using the Centiloid scale (as an alternative measurement unit to SUVR) and CDR-SOB was estimated to be strong. This result was based on data from all 23 trials and 39 contrasts. Further details of the analysis can be found in Supplementary Figure 5 of the Appendix C2.

#### Surrogate relationships for individual treatments

**Figure 3.**
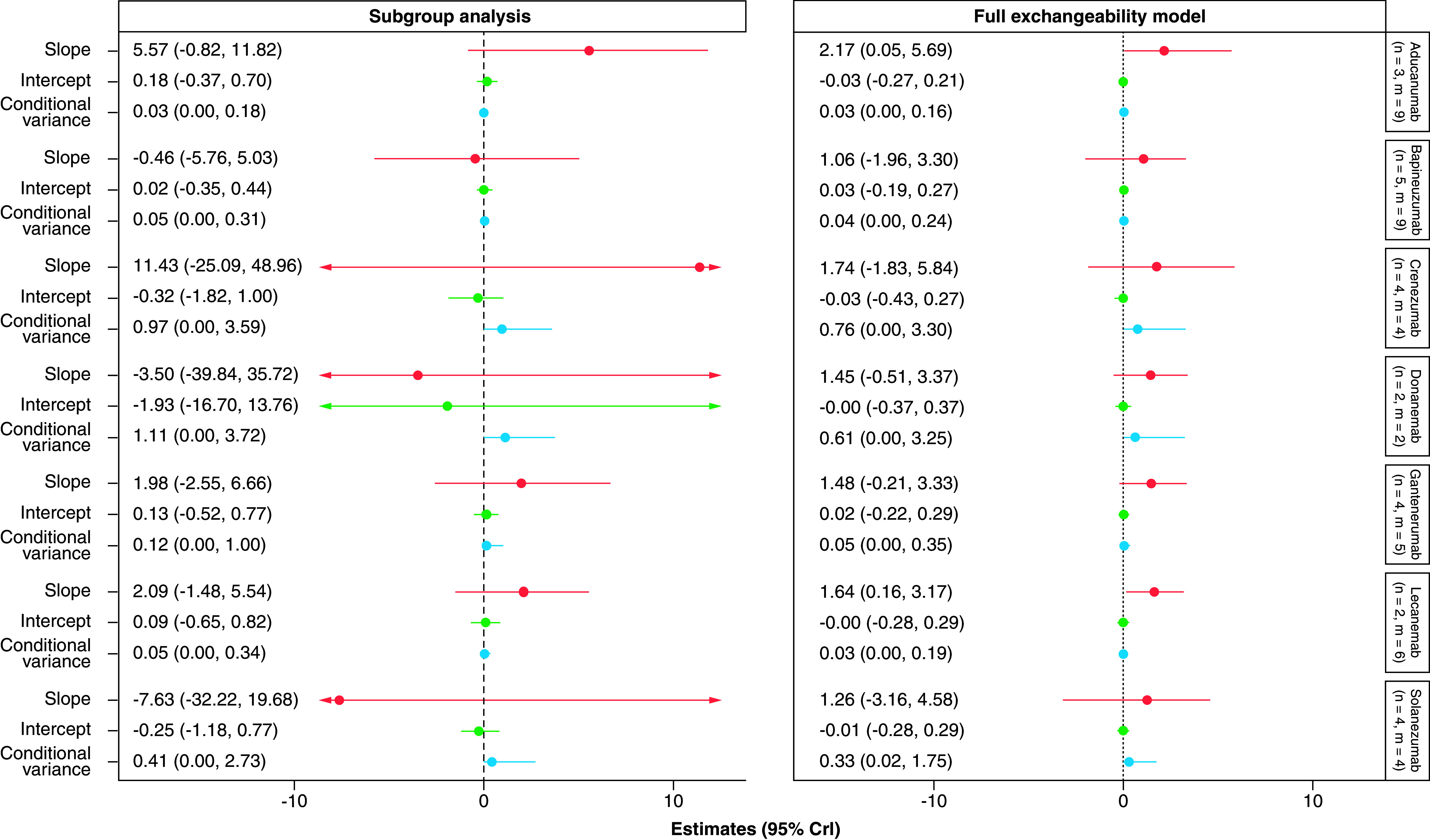
Forest plot of estimates of slope (red), intercept (green) and conditional variance (blue) for amyloid-beta (on the standardized uptake value ratio scale) as a surrogate for Clinical Dementia Ratio – Sum of Boxes using treatment effects on amyloid-beta SUVR at earliest reported time points. Each column represents a different model, and each row corresponds to a different treatment. CDR-SOB: Clinical Dementia Rating-Sum of Boxes; m: Total constrasts included; n: Studies included; SUVR: Standardized uptake value ratio.

The surrogate relationships across trials within individual treatments were also explored. The results using subgroup analysis, and a full exchangeability hierarchical model allowing for information sharing across the treatments, are presented in Figure 3. Each column shows the estimated slope (red), intercept (green) and conditional variance (blue) for each treatment in turn. Given the nature of Bayesian analysis, interpretation of the results should focus on the uncertainty reflected in the CrIs rather than the point estimates themselves.

### Subgroup analyses

The surrogate relationships estimated from the subgroup analyses were weak, with substantial uncertainty indicated by wide CrIs around the association parameters for all individual treatments. For example, the surrogate relationship for lecanemab across treatment effects on SUVR and CDR-SOB was weak with substantial uncertainty, with a slope of 2.09 (95% CrI: -1.48, 5.54) and a conditional variance of 0.05 (95% CrI: 0.00, 0.34). When only data from aducanumab trials were used, the slope was estimated to be 5.57 (95% CrI: -0.82, 11.82) and the conditional variance was estimated to be 0.03 (95% CrI: 0, 0.18), suggesting large uncertainty on the surrogate relationships.

### Hierarchical models

The use of the full exchangeability hierarchical model, through borrowing of information about the surrogate relationship from other treatments, allowed us to estimate the key parameters with much higher precision. For example, the surrogate relationship for lecanemab was stronger when sharing information from trials of other MABs, with a statistically meaningful slope of 1.64 (95% CrI: 0.16, 3.17) and a conditional variance of 0.03 (95% CrI: 0.00, 0.19). Similar results were obtained for aducanumab, where a positive slope of 2.17 (95% CrI: 0.05, 5.69) and a smaller conditional variance of 0.03 (95% CrI: 0.00, 0.16) were obtained when sharing information from other MABs. These results suggest a moderate surrogate relationship between the treatment effect on Aβ measured by SUVR and CDR-SOB for lecanemab and aducanumab, albeit still with a high level of uncertainty around these key parameters.

There was a lack of evidence of a surrogate relationship between the effects on Aβ SUVR and CDR-SOB for all the other drugs, with the 95% CrIs for all estimated slopes including zero and large conditional variances, in particular for crenezumab, donanemab and solanezumab. The use of the information-sharing through the hierarchical model led to the improvement in precision of the estimates for the regression parameters. However, despite the increased precision gained from borrowing information, the surrogate relationships were still uncertain for these individual treatments. While this may partly reflect the small number of trials available for each treatment, results for bapineuzumab were also suboptimal despite having a comparable amount of data, suggesting that the surrogate relationship between Aβ reduction and CDR-SOB may vary across anti-Aβ antibodies.

Similar results across all treatments were obtained from the partial exchangeability model, but with a slightly higher level of uncertainty around the estimates for the slope, intercept and variance. These results are presented in Supplementary Figure 7 of Appendix C3.

Leave-one-out cross-validation, performed to evaluate the predictive value of Aβ using the full exchangeability model, demonstrated 100% coverage, which is likely associated with inflated predicted intervals due to increased uncertainty in the key surrogacy parameters. A forest plot showing the observed effects and predicted effects of CDR-SOB for each study can be found in Supplementary Figure 8 of Appendix C4.

## Discussion

We evaluated Aβ level as a putative surrogate endpoint for clinical function using Bayesian meta-analysis models for surrogate endpoint evaluation. The evidence included in the meta-analysis was extracted from RCTs that reported both the treatment effects on Aβ level and on clinical function. Twenty-three RCTs with 39 treatment contrasts for seven MABs were identified and analyzed collectively. Results from the meta-analysis of 23 RCTs showed that an effect on Aβ was a potential surrogate endpoint for the effect on CDR-SOB when assuming a common surrogate relationship for all included treatments. There was a lack of evidence of a surrogate relationship between treatment effects on Aβ and MMSE based on the meta-analysis of 13 trials. The results from the meta-analysis of 20 RCTs suggested that the surrogate relationship between treatment effects on Aβ and ADAS-Cog was relatively weak.

Recently, results from Pang *et al.* [17], Ackley *et al.* [18] and Wang *et al.* [15] also suggested a significant association between the Aβ reduction and CDR-SOB improvement, consistent with our results. Our meta-analysis combined all available information related to treatment effects on Aβ and clinical function from 23 RCTs using a Bayesian surrogate evaluation model, providing a more robust estimate of the association between the effects on Aβ and the effects on clinical function. Two earlier meta-analyses [16,17] found no association between Aβ reduction and MMSE improvement based on published antibody data, which aligns with our results. Evidence related to treatment effects on MMSE was limited; only 13 of the 23 identified trials reported treatment effect on MMSE, most of which did not achieve a big reduction in Aβ. Also, MMSE has been found to have low sensitivity to cognitive deterioration [51]. These factors may have contributed to the parameters of surrogate relationship obtained with high uncertainty.

The surrogate relationship between treatment effects on Aβ level and change in ADAS-Cog was found to be suboptimal with statistically meaningful slope but moderate variance. Among the 20 included RCTs, four different variants of questionnaires were used to measure ADAS-Cog, which may have introduced additional heterogeneity and, therefore, additional uncertainty of the results. Similarly, results from Pang *et al.* [17] also showed a significant effect of reduction on ADAS-Cog with a wide confidence interval.

Several factors may have contributed to the differences in surrogate relationships across clinical outcomes. From a statistical perspective, our review identified fewer studies reporting outcomes on MMSE and ADAS-cog; limited data result in increased uncertainty in the estimates, potentially contributing to weaker or uncertain surrogate relationships. From a clinical perspective, different outcome measures assess distinct cognitive and functional domains and vary in their sensitivity to change. For instance, MMSE has been shown to have limited sensitivity in detecting subtle cognitive deterioration [51]. Consequently, the choice of outcome measures can influence the surrogacy patterns.

We conducted subgroup analysis to evaluate the surrogate relationships between treatment effects on Aβ and CDR-SOB for individual treatments. The resulting estimates were highly uncertain, largely due to a small number of trials. With the use of Bayesian hierarchical model, individual surrogacy parameters were estimated with much improved precision by borrowing information from trials on other treatments. Statistically meaningful slopes were estimated for aducanumab and lecanemab when allowing for borrowing of information, suggesting a potential surrogate relationship for the two treatments. There was a lack of meaningful improvement in surrogate relationship for other treatments. While this may partly reflect the small number of trials available for each treatment, the results were also suboptimal for Bapineuzumab, despite that the amount of data were comparable if not exceeding the quantity for lecanemab and aducanumab. This suggests that the strength of the surrogate relationship between the effects on Aβ and CDR-SOB may vary across anti-Aβ MABs. Biological differences of the anti-Aβ drugs may also influence how Aβ reduction translates into clinical benefit, and surrogate relationships may not necessarily hold for new therapies.

One limitation of our study was existence of missing data on the treatment effects on Aβ measured on SUVR scale. We used mapping equations to impute the Aβ measurements for six studies that did not report the effect on the SUVR scale but reported Aβ on the Centiloid scale instead. The mapping equations were applied based on the tracer used for Aβ PET imaging. For trials in which multiple tracers were used, the conversion was done using the average of the mapping equations. While the mapping equations allow us to incorporate all the relevant evidence in the analysis, this approach may have also introduced uncertainty into the results. To address this concern, a sensitivity analysis excluding imputed estimates was performed and the results provided in Supplementary Figure 6 of Appendix C2 suggest a moderate surrogate relationship despite having a smaller number of studies. Additionally, there was heterogeneity in the follow-up time across studies. A sensitivity analysis to the length of the follow-up time was also carried out, which showed similar results as the main analysis. Furthermore, the use of different tracers measuring PET Aβ levels across studies may have contributed to additional between-study heterogeneity of the effects on Aβ and, as a result, increased uncertainty in estimating key surrogacy parameters.

The trials identified by Jeremic *et al.* formed the primary evidence base for our research. Their analysis found no evidence of publication bias, supported by symmetric funnel plots and non-significant Egger’s test results for all three outcomes. Although unpublished negative studies in AD cannot be fully excluded, the available data suggest publication bias is unlikely to have influenced our conclusions.

Minimal clinically important difference is the smallest change in a treatment outcome that an individual would identify as important. The minimal clinically important difference framework is helpful in determining whether observed changes translate into meaningful patient benefits. Future research should investigate whether changes in Aβ can lead to clinically meaningful improvements in clinical outcomes.

Previous research has found associations between Aβ reduction, ARIA rate, the ε4 allele of the Apolipoprotein E gene (APOE4) and clinical efficacy [15], suggesting that the proportion of APOE4 carriers and the presence of ARIA may need to be considered when assessing surrogate endpoints. Further research is needed to investigate their potential influence on the surrogate relationship in greater depth. The benefit of the Bayesian hierarchical models may have been limited for treatments with small number of trials, for example, donanemab. Data from a larger number of trials would be required to fully assess the surrogate relationships across other anti-Aβ MABs.

Our findings suggest that Aβ reduction could potentially serve as a surrogate endpoint in clinical trials, thereby accelerating the evaluation of novel AD therapies. By using Aβ reduction as an early indicator of clinical efficacy, researchers may be able to speed up the drug development processes and reduce the time required to bring new therapies to the market. However, it is important to recognize that while Aβ reduction is associated with clinical improvement overall; it does not guarantee such an improvement, particularly in individual treatments including therapies developed in the future. Relying solely on Aβ as a surrogate endpoint may overlook other relevant pathophysiological processes contributing to AD. Aβ reduction should be viewed as part of a broader strategy to understand AD.

## Summary points

- Amyloid-beta (Aβ) was evaluated as a surrogate endpoint for clinical outcomes in Alzheimer’s disease (AD).
- Data from 23 trials of anti-Aβ monoclonal antibodies (MABs) were identified.
- Bayesian meta-analytic model was used to evaluate trial-level surrogacy across all MABs and for each individual treatment.
- Aβ appears to be a good surrogate endpoint for Clinical Dementia Rating Scale-Sum of Boxes (CDR-SOB) when evaluated across all MABs.
- The surrogate relationships for individual treatments were uncertain.
- The use of Bayesian information-sharing method revealed moderate surrogacy for lecanemab and aducanumab.
- Whether the surrogate relationship would hold for new treatments not included in this analysis remains to be assessed.
- The understanding of surrogacy patterns in AD could provide valuable insight for health technology assessment decision-makers.

## Supplementary Material


