# Supplementary material for: Evaluating amyloid-beta as a surrogate endpoint in trials of anti-amyloid-beta drugs in Alzheimer’s disease: a Bayesian meta-analysis
Source: J Comp Eff Res. 2025 Dec 2;15(1):e250095. doi: 10.57264/cer-2025-0095 (PMC12711098; doi:10.57264/cer-2025-0095)
Supplement: Supplementary file 1 [file cer-15-250095-s1.docx]

Supplementary materials to “Evaluating amyloid-beta as a surrogate endpoint in trials of anti-amyloid-beta drugs in Alzheimer’s disease: a Bayesian meta-analysis”

Appendix A. Conversion of data on Aβ PET between Centiloid and SUVR scales

In trials assessing anti-Amyloid-beta (Aβ) drugs, the treatment effect is commonly assessed in terms of the reduction in Aβ volume within the brain measured via positron emission tomography (PET) imaging. The PET images are inspected to quantify the Aβ volume as a standardized uptake value ratio (SUVR). PET imaging involves the injection of a radioactive tracer which may differ from trial to trial, hindering the comparison of SUVR values across trials. To facilitate cross-trial comparisons, methods have been proposed to convert SUVR values to a standard Centiloid scale ^1^.

In our evidence base, the majority of trials reported data on the SUVR scale, but there were a few trials which reported data on the Centiloid scale only. To make the most efficient use of this evidence, we converted these data from the Centiloid scale to the SUVR scale. A number of studies have implemented the conversion from the SUVR scale to the Centiloid scale based on the tracer used for Aβ PET imaging; florbetapir ^2^, florbetaben^3^, flutemetamol ^4^. We applied the regression equations reported in these studies to make the conversion of data from the Centiloid scale to the SUVR scale, according to the tracer used in each trial. Specifically, we used the following equations:

- Florbetapir: Centiloid = 183 x SUVR – 177

- Florbetaben: Centiloid = 153.4 x SUVR -154.9

- Flutemetamol: Centiloid = 116 x SUVR – 113.9

These equations were originally applied to convert SUVR values recorded at a particular point in time. In our evidence base, the data are reported as the change from baseline so we apply each equation without the intercept terms (since these terms would cancel out when applying the equation to data at each time point, i.e., at baseline and follow-up). For trials in which the multiple tracers were used (e.g., florbetapir and florbetaben), we make the conversion using the average of the regression equations (i.e., averaging the coefficients).

Appendix B. Literature review

Three studies include the effect of anti-Aβ drugs on both PET Aβ and clinical outcome measures (ADAS-Cog, CDR-SOB and MMSE) ^5–7^. Although the results of these meta-analyses showed an overall significant effect in reducing Aβ, the effect on the clinical outcome measures varied across the meta-analyses. Avgerinos *et al.* ^5^ found a small positive effect of anti-Aβ drugs on both ADAS-Cog and MMSE, but no effect was evident on CDR-SOB based on 17 studies. Lacorte *et al.* ^6^ identified and found a negative effect of anti-Aβ drugs on CDR-SOB after performing a synthesis. Lyu *et al.* ^7^ found significant effects on ADAS-Cog, CDR-SOB and MMSE.

There were six meta-analyses ^8–13^ focusing on different clinical outcomes. Jeremic et al. ^8^ found a positive effect of anti-Aβ drugs on ADAS-Cog, MMSE, and CDR-SOB. Richard *et al.* ^9^ found a lack of evidence of an effect on ADAS-Cog based on six trials. Fernandez *et al.* ^10^ found a small but statistically significant effect on ADAS-Cog and MMSE based on 12 RCTs. Villain *et al.* ^11^ found a statistically significant effect on ADAS-Cog and CDR-SOB based on evidence from 4 RCTs demonstrating high level of amyloid clearance, but no effect was found on MMSE. After conducting a Bayesian random effects meta-analysis, Teipel *et al.* ^12^ found a moderate to small but statistically meaningful effect (with 95% credible intervals excluding zero) on CDR-SOB based on eight studies. Holdridge *et al.* ^13^ found a statistically significant effect across various functional outcome measures based solely on three phase III studies of solanezumab.

Table S1: Characteristics of included clinical trials

| **Study** | **Registration number** | **Drugs** | **Study duration** | **Dose** | **Number of patients** | **MCI due to AD or Prodromal AD (%)** | **Mild AD (%)** |
| --- | --- | --- | --- | --- | --- | --- | --- |
| EMERGE | NCT02484547 | Aducanumab | 76 weeks | Placebo | 548 | 81 | 19 |
|  |  |  |  | 3mg/kg or 6mg/kg | 543 | 83 | 17 |
|  |  |  |  | 6mg/kg or 10mg/kg | 547 | 80 | 20 |
| ENGAGE | NCT02477800 | Aducanumab | 76 weeks | Placebo | 545 | 81 | 19 |
|  |  |  |  | 3mg/kg or 6mg/kg | 547 | 80 | 20 |
|  |  |  |  | 6mg/kg or 10mg/kg | 555 | 80 | 20 |
| PRIME | NCT01677572 | Aducanumab | 54 weeks | Placebo | 48 | 46 | 54 |
|  |  |  |  | 1 mg/kg | 31 | 32 | 68 |
|  |  |  |  | 3 mg/kg | 32 | 44 | 56 |
|  |  |  |  | 6 mg/kg | 30 | 40 | 60 |
|  |  |  |  | 10 mg/kg | 32 | 41 | 59 |
|  |  |  |  | Titration | 23 | 57 | 43 |
| BAN2401-G000-201 | NCT01767311 | Lecanemab | 18 months | Placebo | 238 | 65 | 35 |
|  |  |  |  | 2.5 mg/kg biweekly | 52 | 65 | 35 |
|  |  |  |  | 5 mg/kg monthly | 48 | 69 | 31 |
|  |  |  |  | 5 mg/kg biweekly | 89 | 58 | 42 |
|  |  |  |  | 10 mg/kg monthly | 246 | 68 | 32 |
|  |  |  |  | 10 mg/kg biweekly | 152 | 59 | 41 |
| Clarity AD | NCT03887455 | Lecanemab | 18 months | Placebo | 875 | 62.2 | 37.8 |
|  |  |  |  | 10 mg/kg biweekly | 859 | 61.5 | 38.5 |
| Trailblazer-ALZ | NCT03367403 | Donanemab | 72 weeks | Placebo | 126 | prodromal AD or mild AD with dementia | |
|  |  |  |  | 700 mg for the first three doses and 1400 mg thereafter | 131 |  |  |
| Trailblazer-ALZ2 | NCT04437511 | Donanemab | 72 weeks | Placebo | 876 | 15.7 | 84.3 |
|  |  |  |  | 700 mg for the first three doses and 1400 mg thereafter | 860 | 17 | 83 |
| SCarlet RoAD | NCT01224106 | Gantenerumab | 2 years | Placebo | 266 | prodromal AD | |
|  |  |  |  | 105 mg every 4 weeks | 271 |  |  |
|  |  |  |  | 225 mg every 4 weeks | 260 |  |  |
| Graduate I | NCT03444870 | Gantenerumab | 116 weeks | Placebo | 485 | 54.2 | 45.8 |
|  |  |  |  | 510mg biweekly | 499 | 55.1 | 44.9 |
| Graduate II | NCT03443973 | Gantenerumab | 116 weeks | Placebo | 477 | 55.8 | 44.2 |
|  |  |  |  | 510mg biweekly | 498 | 54 | 46 |
| Bapineuzumab 301 | NCT00575055 | Bapineuzumab | 78 weeks | Placebo | 432 | mild to moderate AD | |
|  |  |  |  | 0.5 mg/kg | 658 |  |  |
| Bapineuzumab 302 | NCT00574132 | Bapineuzumab | 78 weeks | Placebo | 493 | mild to moderate AD | |
|  |  |  |  | 0.5 mg/kg | 314 |  |  |
|  |  |  |  | 1 mg/kg | 307 |  |  |
| Study 3000 | NCT00667810 | Bapineuzumab | 78 weeks | Placebo | 431 | mild to moderate AD | |
|  |  |  |  | 0.5 mg/kg | 650 |  |  |
| Study 3001 | NCT00676143 | Bapineuzumab | 78 weeks | Placebo | 328 | mild to moderate AD | |
|  |  |  |  | 0.5 mg/kg | 255 |  |  |
|  |  |  |  | 1 mg/kg | 253 |  |  |
| Brody 2016 | NCT01254773 | Bapineuzumab | 48 weeks | Placebo | 36 | mild to moderate AD | |
|  |  |  |  | 2 mg/month | 37 |  |  |
|  |  |  |  | 7 mg/month | 36 |  |  |
|  |  |  |  | 20 mg/month | 37 |  |  |
| BLAZE | NCT01397578 | Crenezumab | 68 weeks | Placebo | 13 | mild to moderate AD | |
|  |  |  |  | 300 mg | 26 |  |  |
| BLAZE | NCT01397578 | Crenezumab | 68 weeks | Placebo | 17 | mild to moderate AD | |
|  |  |  |  | 15 mg/kg | 35 |  |  |
| CREAD | NCT02670083 | Crenezumab | 100 weeks | Placebo | 409 | 42.8 | 57.2 |
|  |  |  |  | 60 mg/kg | 404 | 42.3 | 57.7 |
| CREAD2 | NCT03114657 | Crenezumab | 100 weeks | Placebo | 399 | 47.6 | 52.4 |
|  |  |  |  | 60 mg/kg | 407 | 48.6 | 51.4 |
| EXPEDITION3 | NCT01900665 | Solanezumab | 76 weeks | Placebo | 1072 | mild dementia due to AD | |
|  |  |  |  | 400 mg | 1057 |  |  |
| EXPEDITION EXT | NCT01127633 | Solanezumab | 104 | Placebo | 723 | mild dementia due to AD | |
|  |  |  |  | 400 mg | 734 |  |  |
| A4 | NCT02008357 | Solanezumab | 240 weeks | Placebo | 583 | Unknown | |
|  |  |  |  | 1600 mg | 564 |  |  |
| DIAN-TU-001 | NCT04623242 | Gantenerumab | 4-7 years | Placebo | 40 | dominantly inherited AD | |
|  |  |  |  | Solanezumab 400 mg to 1600 mg | 50 |  |  |
|  |  |  |  | Gantenerumab 225 mg to 1200 mg | 52 |  |  |

MCI, mild cognitive impairment; AD, Alzheimer’s disease.

Appendix C. Further Results

Appendix C1. Cross validations and predictions for the analysis of surrogate relationships across all trials of MABs

Leave-one-out cross-validation was performed to evaluate the predictive value of Aβ as a surrogate endpoint for CDR-SOB. The Daniels and Hughes model showed a good coverage with 95% of the predicted intervals including the observed estimates of the effects on CDR-SOB. The average absolute difference between the observed and predicted effect estimates was 0.32, and the average ratio of the width of intervals between the predicted and observed treatment effect was 1.24. A forest plot showing the observed effects and predicted effects of CDR-SOB for each study are presented in Figure S1.


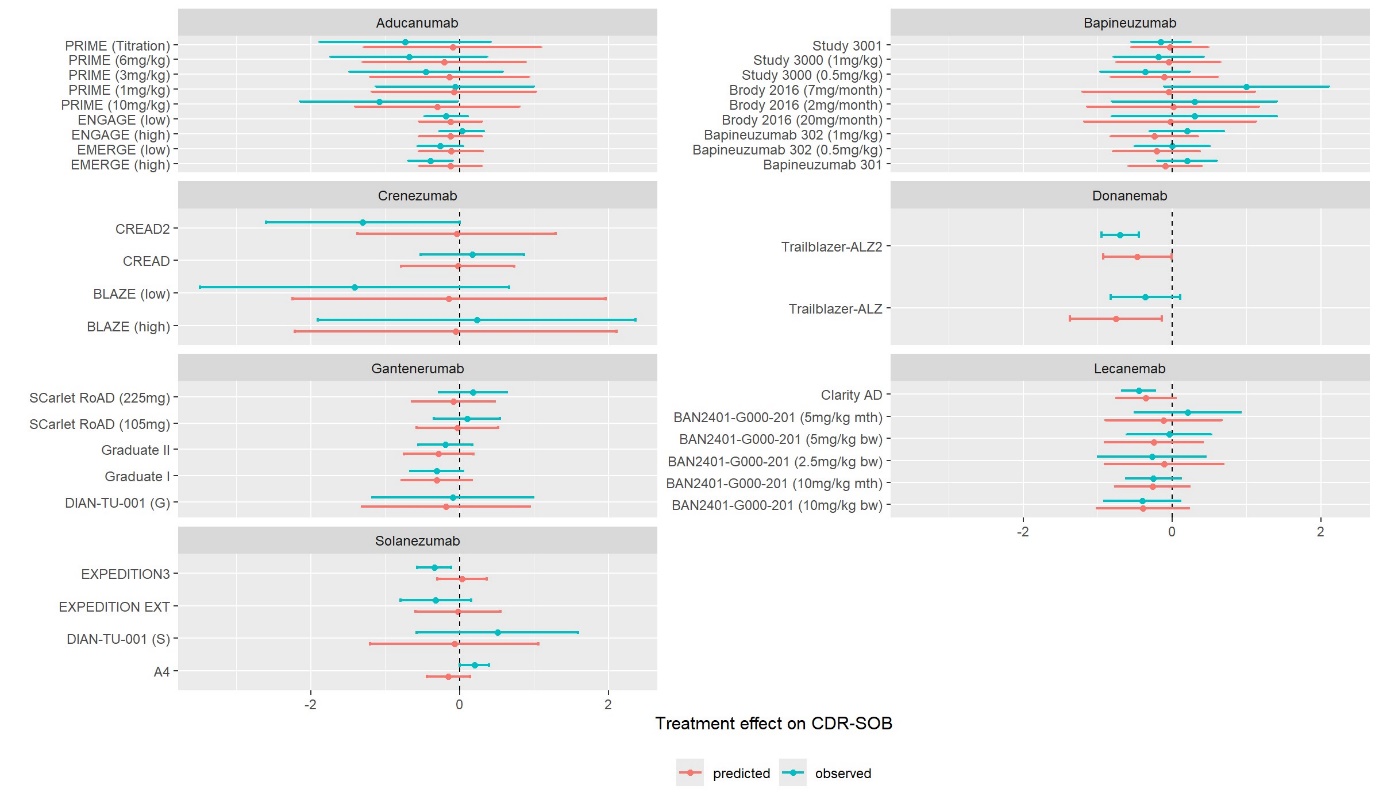
Figure S1. Forest plot illustrating the observed treatment on Clinical Dementia Rating – Sum of Boxes (CDR-SOB) and the corresponding predicted effect on CDR-SOB using Daniels and Hughes model.

Appendix C2. Further analyses for evaluating surrogate relationship across all trials of MABs

Analyses were also conducted to explore the overall surrogate relationship between treatment effects on Aβ SUVR and other clinical outcomes. The surrogate relationship between treatment effects on Aβ SUVR and ADAS-Cog was estimated to be weak, with slope 3.71 (95% CrI: 1.44, 6.05) and 0.06 (95% CrI: 0, 0.28). This analysis is based on data extracted from 20 studies (31 treatment contrasts) that have collected both treatment effects on Aβ and ADAS-Cog. Among the 20 included studies, five studies reported ADAS-Cog11, two studies reported ADAS-Cog12, eight studies reported ADAS-Cog13 and five studies reported ADAS-Cog14. 13 studies with 22 contrasts reported both treatment effects on Aβ and MMSE. The surrogate relationship between treatment effects on Aβ SUVR and MMSE was uncertain, with slope -1.14 (95% CrI: -2.84, 0.49) and conditional variance 0.06 (95% CrI: 0, 0.23). The bubble plot and regression line are presented in Figure S2 and Figure S3 respectively.

When the treatment effects were reported at the same time, the surrogate relationship between Aβ SUVR and CDR-SOB was also strong, with slope 0.93 (95% CrI: 0.4, 1.47) and conditional variance 0.02 (95% CrI: 0, 0.05). The bubble plot and regression line are presented in Figure S4. In some trials, Aβ levels were measured on a centiloid scale instead of the SUVR scale. The surrogate relationship between treatment effects on Aβ using centiloid measure and CDR-SOB was found to be strong, with slope 0.01 (95% CrI: 0, 0.01) and conditional variance 0.02 (95% CrI: 0, 0.05). The bubble plot and regression line are presented in Figure S5. Six studies were identified with both treatment effects on Aβ centiloids and CDR-SOB reported. Despite the limited number of studies included, the surrogate relationship across the six included studies was found to be moderate with slope 0.01 (95% CrI: 0, 0.02) and conditional variance 0.05 (95% CrI: 0, 0.35). The bubble plot and regression line are presented in Figure S6.


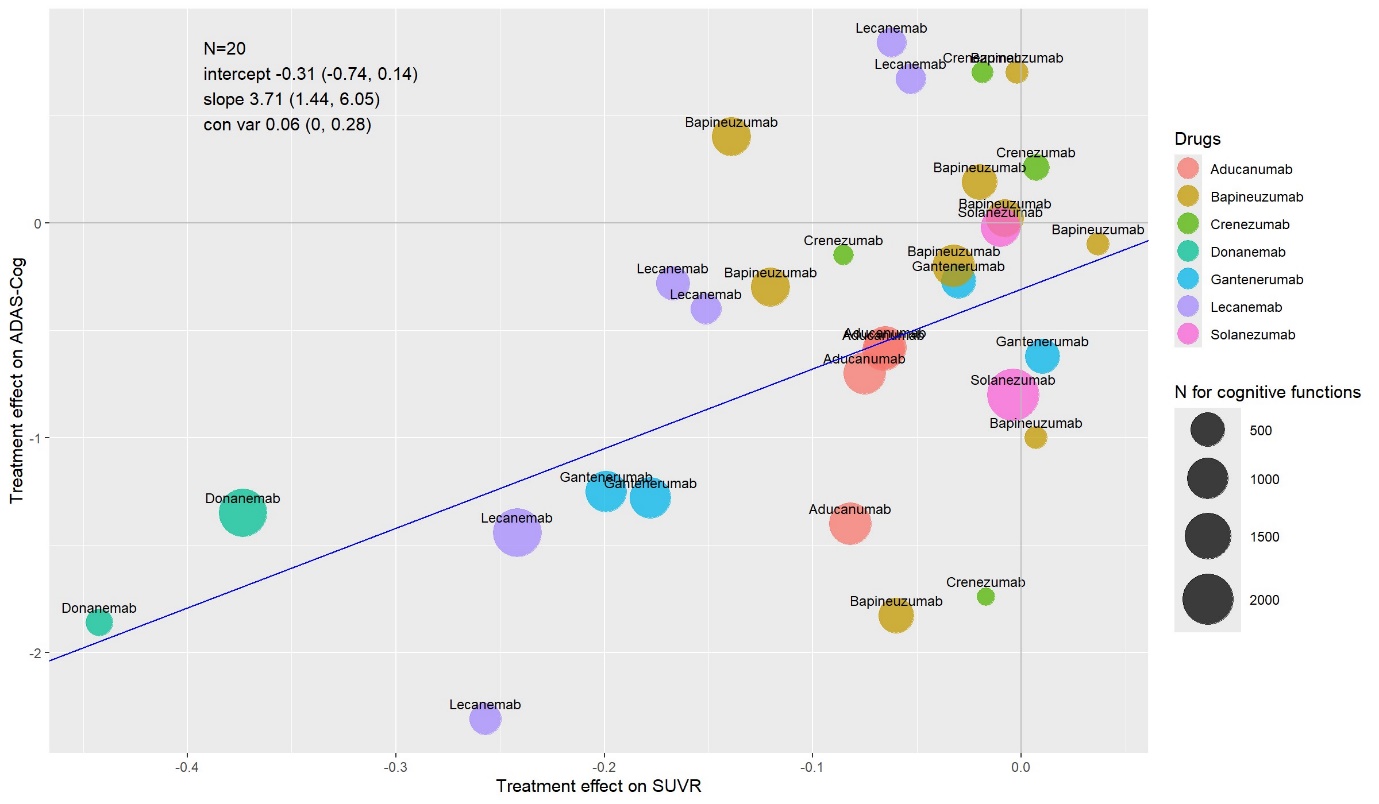


Figure S2. Bubble plot of the surrogate relationship between treatment effects on Aβ standardised uptake value ratio (SUVR) and Alzheimer’s Disease Assessment Scale--Cognitive Subscale (ADAS-Cog) with treatment effects on Aβ reported at earlier time points. The mean (95% credible intervals) of intercept, slope and conditional variance were obtained from Daniels and Hughes model. The bubble size corresponds to the number of patients with measured cognitive functions.


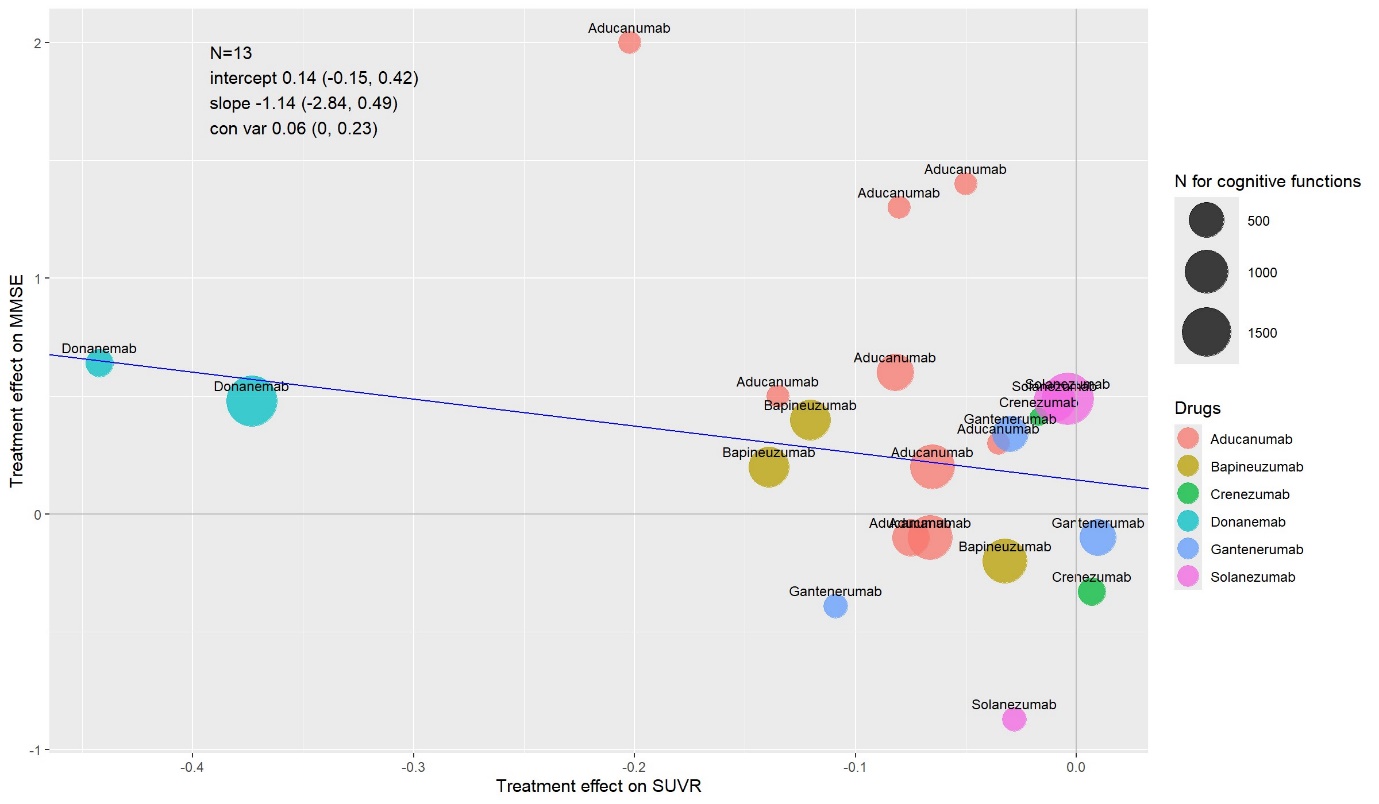


Figure S3. Bubble plot of the surrogate relationship between treatment effects on Aβ standardised uptake value ratio (SUVR) and Mini Mental State Examination (MMSE) with treatment effects on Aβ reported at earlier time points. The mean (95% credible intervals) of intercept, slope and conditional variance were obtained from Daniels and Hughes model. The bubble size corresponds to the number of patients with measured cognitive functions.


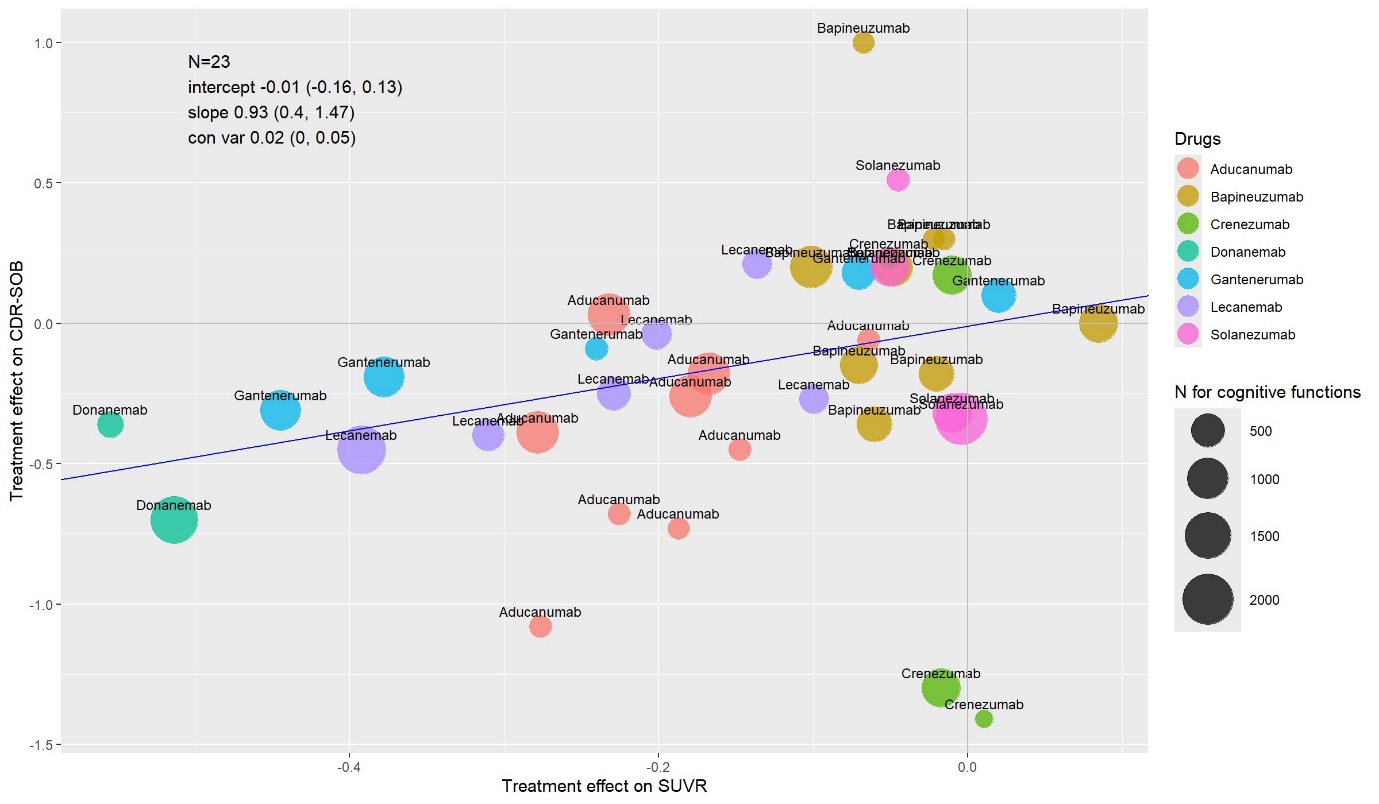


Figure S4. Bubble plot of the overall surrogate relationships with treatment effects on Aβ standardised uptake value ratio (SUVR) and Clinical Dementia Rating – Sum of Boxes (CDR-SOB) reported at the same time. The mean (95% credible intervals) of intercept, slope and conditional variance were obtained from Daniels and Hughes model. The bubble size corresponds to the number of patients with measured cognitive functions.


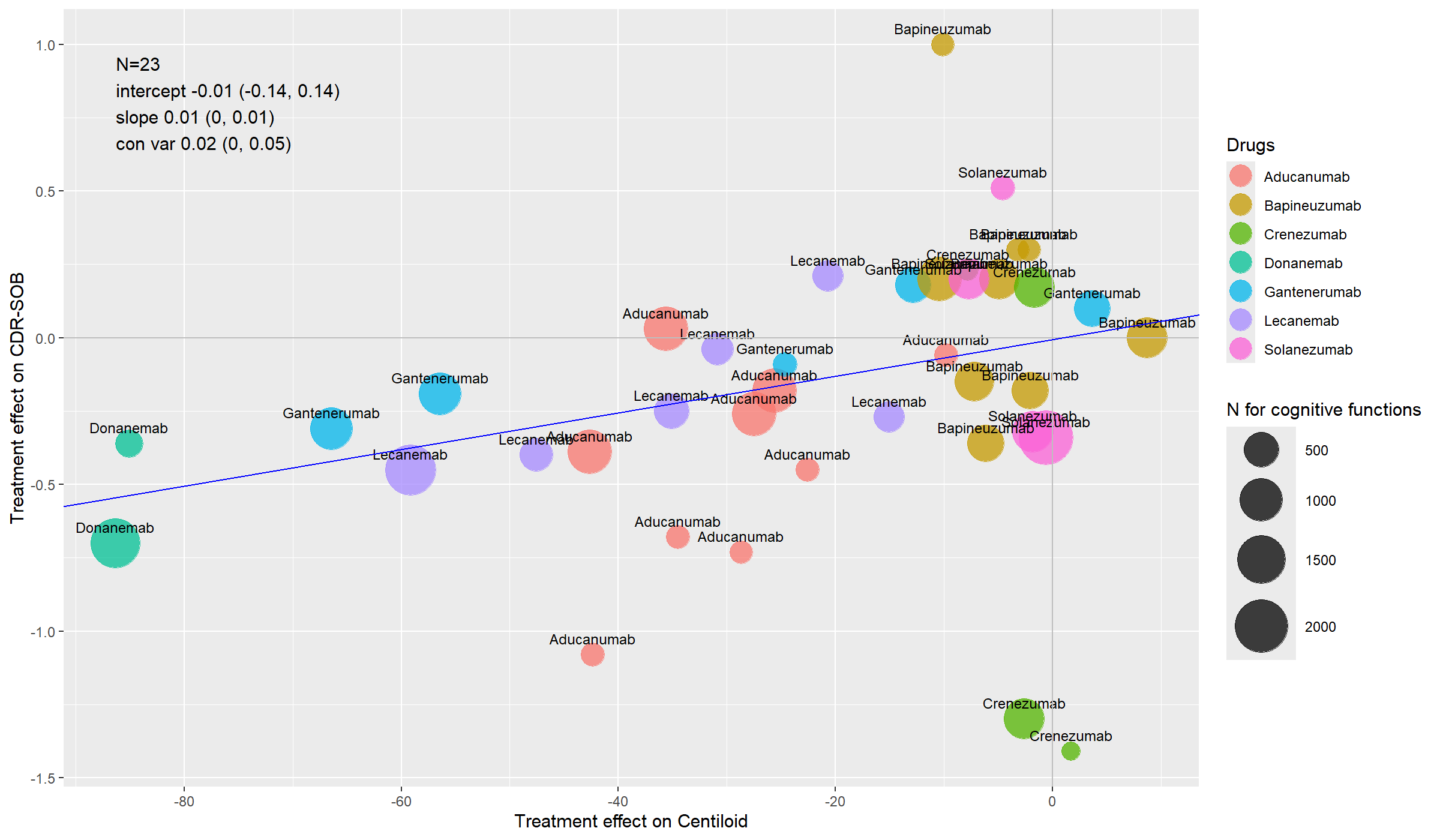


Figure S5. Bubble plot of the overall surrogate relationships with treatment effects on Aβ centiloids (with converted values) and Clinical Dementia Rating – Sum of Boxes (CDR-SOB) reported at the same time. The mean (95% credible intervals) of intercept, slope and conditional variance were obtained from Daniels and Hughes model. The bubble size corresponds to the number of patients with measured cognitive functions.


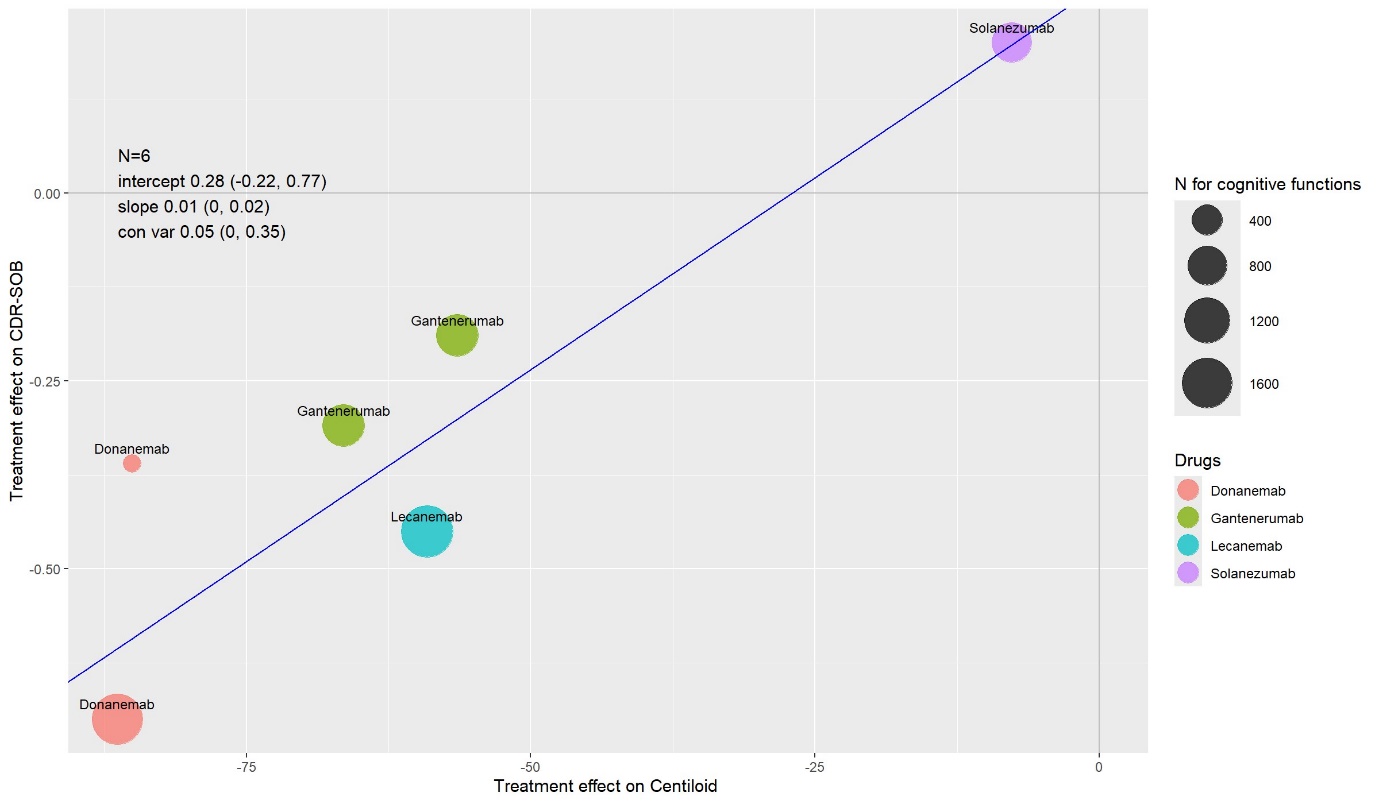


Figure S6. Bubble plot of the overall surrogate relationships with treatment effects on Aβ centiloids (without converted values) and Clinical Dementia Rating – Sum of Boxes (CDR-SOB) reported at the same time. The mean (95% credible intervals) of intercept, slope and conditional variance were obtained from Daniels and Hughes model. The bubble size corresponds to the number of patients with measured cognitive functions.

Appendix C3. Further analyses for evaluating surrogate relationship by treatment

The surrogate relationships for individual treatments were discussed in Section 3.2.1. The results of partial exchangeability model are presented along with the results of subgroup analysis and full exchangeability model in Figure S7.

The partial exchangeability hierarchical model allows for the flexible borrowing of information across treatments, resulting in the estimation of key parameters with less uncertainty. The surrogate relationship for lecanemab from the partial exchangeability hierarchical model was moderate, with the slope 1.71 (95% CrI: 0.08, 3.35) and conditional variance 0.03 (95% CrI: 0, 0.22). The surrogate relationship for all the other drugs was weak with zero included in the credible intervals of slope. For example, for aducanumab, the slope was 2.59 (95% CrI: -0.03, 6.92) and conditional variance was 0.03 (95% CrI: 0, 0.16).

The posterior mean of mixture weights from the partial exchangeability model had an average of 0.83, indicating a high level of borrowing of information. When comparing results from the full exchangeability model with subgroup analyses, the reduction in the width of CrI was 71% (51%-95%) for slope and 28% (7%-65%) for conditional variance. When comparing results from the partial exchangeability model with subgroup analyses, the reduction in the width of CrI was 54% (34%-94%) for slope and 24% (6%-63%) for conditional variance. The reduction is greater for the full exchangeability model as more information was borrowed in the full exchangeability model.


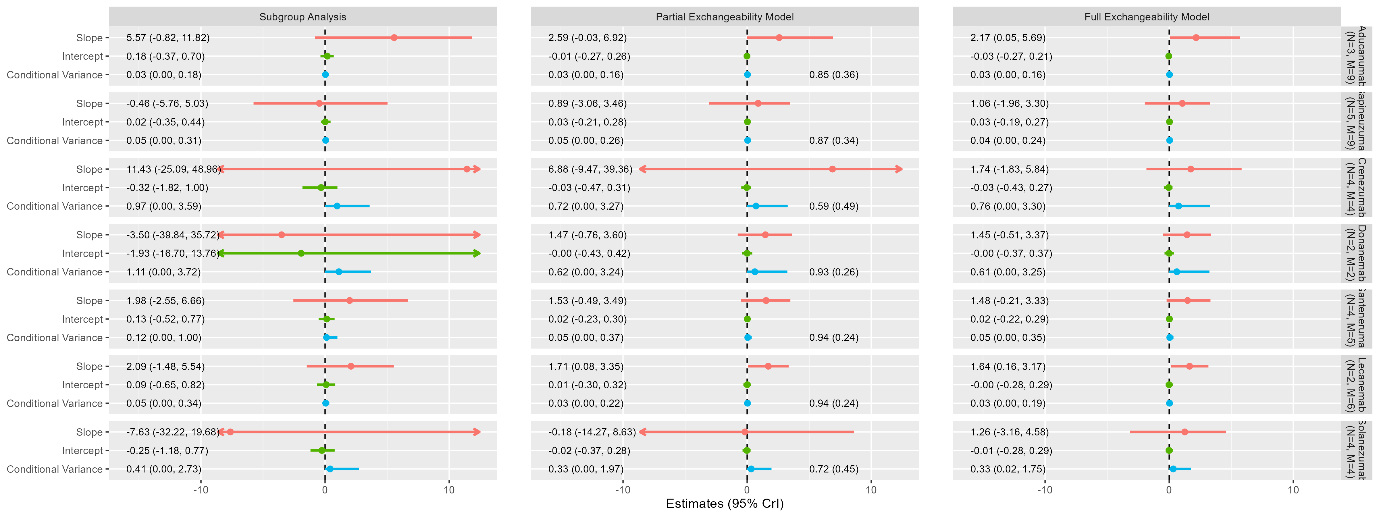


Figure S7. Forest plot of estimates of slope (red), intercept (green) and conditional variance (blue) for Aβ standardised uptake value ratio (SUVR) as a surrogate for Clinical Dementia Rating – Sum of Boxes (CDR-SOB). Each column represents a different model, and each row corresponds to a different treatment. N represents the number of studies included and M represent the total number of contrasts included.

Appendix C4. Cross validations and predictions for the analysis of surrogate relationships for individual treatments

Leave-one-out cross-validation was also performed to evaluate the predictive value of Aβ for CDR-SOB using the hierarchical models. The full exchangeability model demonstrated good coverage in terms of all predicted intervals including the observed effect estimates on CDR-SOB. The average absolute difference between the observed and predicted effect estimates was 0.32, and the average ratio of the width of intervals between the predicted and observed treatment effect was 2.49. A forest plot showing the observed effects and predicted effects of CDR-SOB for each study are presented in Figure S8.


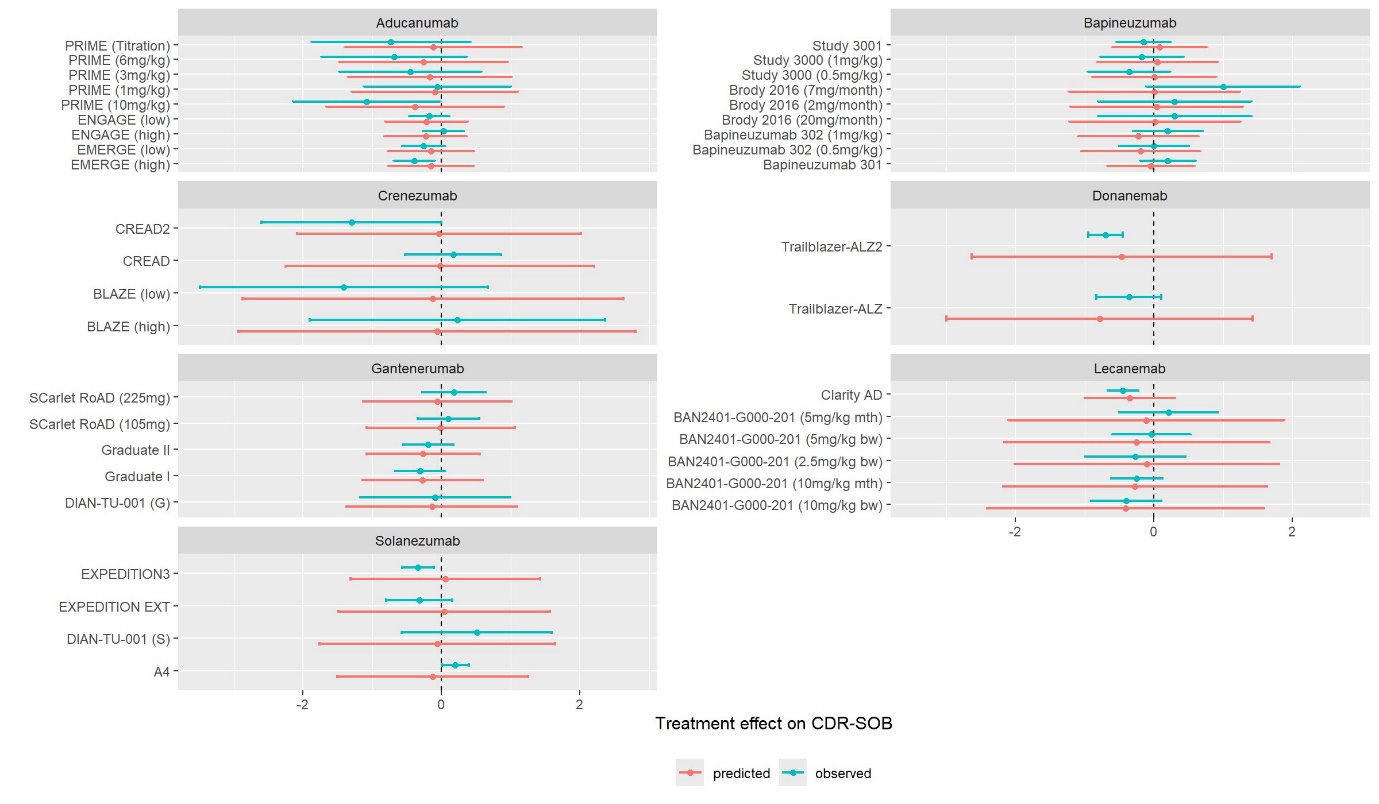
Figure S8. Forest plot illustrating the observed treatment on Clinical Dementia Rating – Sum of Boxes (CDR-SOB) and the corresponding predicted effect on CDR-SOB using full exchangeability model.

References

1. Klunk, W. E. *et al.* The Centiloid Project: Standardizing quantitative amyloid plaque estimation by PET. *Alzheimer’s & Dementia* 11, 1-15.e4 (2015).

2. Navitsky, M. *et al.* Standardization of amyloid quantitation with florbetapir standardized uptake value ratios to the Centiloid scale. *Alzheimer’s & Dementia* 14, 1565–1571 (2018).

3. Rowe, C. C. *et al.* 18F-Florbetaben PET beta-amyloid binding expressed in Centiloids. *Eur J Nucl Med Mol Imaging* 44, 2053–2059 (2017).

4. Hanseeuw, B. J. *et al.* Defining a Centiloid scale threshold predicting long-term progression to dementia in patients attending the memory clinic: an [18F] flutemetamol amyloid PET study. *Eur J Nucl Med Mol Imaging* 48, 302–310 (2021).

5. Avgerinos, K. I., Ferrucci, L. & Kapogiannis, D. Effects of monoclonal antibodies against amyloid-β on clinical and biomarker outcomes and adverse event risks: A systematic review and meta-analysis of phase III RCTs in Alzheimer’s disease. *Ageing Res Rev* 68, 101339 (2021).

6. Lacorte, E. *et al.* Safety and Efficacy of Monoclonal Antibodies for Alzheimer’s Disease: A Systematic Review and Meta-Analysis of Published and Unpublished Clinical Trials. *J Alzheimers Dis* 87, 101–129 (2022).

7. Lyu, D., Lyu, X., Huang, L. & Fang, B. Effects of three kinds of anti-amyloid-β drugs on clinical, biomarker, neuroimaging outcomes and safety indexes: A systematic review and meta-analysis of phase II/III clinical trials in Alzheimer’s disease. *Ageing Research Reviews* 88, 101959 (2023).

8. Jeremic, D., Navarro-López, J. D. & Jiménez-Díaz, L. Efficacy and safety of anti-amyloid-β monoclonal antibodies in current Alzheimer’s disease phase III clinical trials: A systematic review and interactive web app-based meta-analysis. *Ageing Res Rev* 90, 102012 (2023).

9. Richard, E., den Brok, M. G. H. E. & van Gool, W. A. Bayes analysis supports null hypothesis of anti-amyloid beta therapy in Alzheimer’s disease. *Alzheimers Dement* 17, 1051–1055 (2021).

10. Fernandez, P. E. L. & Silva, G. D. Cognitive outcomes of anti-amyloid-β monoclonal antibodies in patients with Alzheimer’s disease: A systematic review and meta-analysis of randomized controlled trials. *Alzheimer’s & Dementia* 17, e057778 (2021).

11. Villain, N., Planche, V. & Levy, R. High-clearance anti-amyloid immunotherapies in Alzheimer’s disease. Part 1: Meta-analysis and review of efficacy and safety data, and medico-economical aspects. *Rev Neurol (Paris)* 178, 1011–1030 (2022).

12. Teipel, S. J., Temp, A. G. M. & Lutz, M. W. Bayesian meta-analysis of phase 3 results of aducanumab, lecanemab, donanemab, and high-dose gantenerumab in prodromal and mild Alzheimer’s disease. *Alzheimers Dement (N Y)* 10, e12454 (2024).

13. Holdridge, K. C., Yaari, R., Hoban, D. B., Andersen, S. & Sims, J. R. Targeting amyloid β in Alzheimer’s disease: Meta-analysis of low-dose solanezumab in Alzheimer’s disease with mild dementia studies. *Alzheimers Dement* 19, 4619–4628 (2023).
